# “All sorts of colours of emotions”: Ambulance call-handlers’ perceptions of the barriers to CPR in out-of-hospital cardiac arrest

**DOI:** 10.1016/j.resplu.2025.100904

**Published:** 2025-02-15

**Authors:** Barbara Farquharson, Marie Johnston, Rosaleen O’Brien, Gareth Clegg

**Affiliations:** a Centre for Healthcare and Community Research (CHeCR), University of Stirling, FK9 4LA, United Kingdom; b University of Aberdeen, United Kingdom; c Independent Researcher, United Kingdom; d University of Edinburgh, United Kingdom

**Keywords:** Cardiopulmonary Resuscitation, Cardiac arrest, Out of hospital, Emergency Medical Service Communication Systems

## Abstract

**Aim:**

To explore call-handlers’ perceptions of the main barriers to achieving CPR during emergency calls to the ambulance service.

**Methods:**

Thirty purposively sampled call-handlers, working in seven UK ambulance dispatch centres, participated in semi-structured qualitative interviews designed to explore their experiences of providing CPR instructions and their perceptions of the most common barriers to initiation of CPR.

**Results:**

Participants (20F 9 M 1non-binary), aged 21–57 years, with varied length of experience (6mths −25 yrs), self-reported confidence (3–10/10), experience of NHS Pathways and MPDS, described providing CPR calls typically once per shift, with most call-handlers reporting barriers to CPR in most calls.

The barriers to initiating CPR most commonly identified by call-handlers were the strong emotions experienced by callers; physical issues relating to the caller, patient and situation; uncertainty about whether CPR was required, particularly uncertainty about breathing and caller concerns about doing harm.

Participants described many overlapping issues, making each call a unique challenge. They also provided insights into the complexities of ambiguous situations such as those encountered by carers and care-homes, DNACPR issues, as well as facilitating factors.

**Conclusion:**

Call-handlers identified barriers to CPR that echo those identified via other study methods plus provide additional insights into areas not readily addressed by current protocols. Call-handlers’ perspectives may be helpful in identifying priority areas for protocol refinement and ways to improve the efficacy of CPR instructions.

## Introduction

Providing bystander cardiopulmonary resuscitation (CPR) in out-of-hospital cardiac arrest increases survival but unfortunately is often not achieved prior to the arrival of emergency services.^1^, ^2^, ^3^ In efforts to improve rates of CPR, and thus survival from OHCA, dispatcher-assisted CPR (DA-CPR) or telephone/telecommunicator-assisted CPR (T-CPR), where trained call-takers provide real-time instructions to callers about how to perform CPR, has been widely implemented globally.^4^, ^5^ DA-CPR is effective in increasing bystander provision of CPR^6^, ^7^, ^8^, ^9^ and survival from OHCA.^6^, ^10^, ^11^, ^12^ However, CPR is not always delivered,^13^, ^14^, ^15^ and time to initiation of CPR can vary widely, even with highly-protocolised DA-CPR instruction.^16^

Exploration of barriers to CPR in the dispatcher-assisted context has been limited, but high-quality evidence has started to accumulate in recent years.^17^, ^18^, ^19^, ^20^, ^21^ Most existing evidence involves analysis of data from registry data and call-recordings^18^, ^19^, ^20^, ^21^, ^22^, ^23^, ^24^, ^25^, ^26^, ^27^, ^28^ and has identified communication issues,^19^, ^20^, ^22^ emotion^19^, ^21^, ^23^, ^24^ and physical challenges^19^, ^21^, ^25^, ^26^, ^28^ as common barriers to achieving DA-CPR. Potential means to improve DA-CPR are also beginning to emerge.^14^, ^29^, ^30^ Few studies have explored the perspectives of call-handlers themselves, a group who are likely to possess valuable tacit knowledge.^31^ Tacit knowledge is created through recurring clinical work whereby individuals, motivated to improve their effectiveness, intuitively adjust their actions to obtain optimal results.^32^ We consider it essential to try to explore the tacit knowledge of those who perform the uniquely critical role of providing CPR instructions over the telephone in order that it be used to inform interventions to improve effectiveness.

Aim: to explore call-handlers’ perceptions of the main barriers to achieving CPR during emergency calls to the ambulance service.

## Methods

### Design

A qualitative semi-structured interview study of staff working in UK ambulance dispatch centres whose role involves providing CPR instructions (job titles vary but subsequently referred to as ‘call-handlers’).

### Study setting

UK Ambulance Medical Dispatch Centres (EMDCs).

### Participants

UK call-handlers took part in the study.

Participants were eligible if they worked in a UK EMDC in a role that involved providing CPR instructions to callers. There were no exclusion criteria.

### Sampling and sample size

We selected participants from seven EMDCs – a number adequate to represent each of the four countries of the UK and to provide diversity in terms of geographical location, urban/rural, size (population served), dispatch software used (MPDS and Pathways), published outcomes for Return of Spontaneous Circulation in OHCA^33^ and Care Quality Commission Rating^34^ (see Table 1 for details). A purposive sample of 30 call-handlers was selected from the pool of 64 who volunteered for the study – a number adequate to achieve diversity in terms of, age, gender, years of experience and confidence in providing CPR instructions. (Experiences were remarkably consistent and so no additional sampling to achieve saturation was required) (see Table 2).

**Table 1:** Ambulance service characteristics.

	Population	Software	Geography
Ambulance Service A	3–6 million	MPDS	mix of rural & cities
Ambulance Service B	3–6 million	Pathways	mostly rural
Ambulance Service C	>6 million	MPDS	urban
Ambulance Service D	>6 million	MPDS	mix of rural & cities
Ambulance Service E	<3 million	MPDS	mix of rural & cities
Ambulance Service F	3–6 million	MPDS	mix of rural & cities
Ambulance Service G	<3 million	MPDS	mix of rural & cities

**Table 2:** Participant characteristics.

	Service	Age	Gender	Years of experience	Confidence (0–10)
Participant 101	Ambulance Service F	27	Female	4.5	9
Participant 103	Ambulance Service F	22	Male	1	7
Participant 104	Ambulance Service F	45	Female	0.83	6
Participant 105	Ambulance Service F	27	Female	1	5
Participant 110	Ambulance Service F	57	Female	8.5	9
Participant 113	Ambulance Service A	35	Female	4	10
Participant 114	Ambulance Service A	26	Male	5	9
Participant 118	Ambulance Service A	39	Female	3.5	9
Participant 119	Ambulance Service C	31	Female	2	9
Participant 120	Ambulance Service A	53	Female	13.5	10
Participant 123	Ambulance Service C	35	Female	8	9
Participant 124	Ambulance Service C	23	Male	0.5	3
Participant 126	Ambulance Service C	39	Female	0.5	8
Participant 127	Ambulance Service C	52	Female	3	8
Participant 128	Ambulance Service E	33	Male	8	10
Participant 133	Ambulance Service E	26	Female	1.3	9
Participant 134	Ambulance Service C	34	Female	9	7
Participant 135	Ambulance Service E	32	Male	1	8
Participant 137	Ambulance Service E	36	Female	18.5	10
Participant 140	Ambulance Service D	24	Male	1.5	10
Participant 143	Ambulance Service D	58	Female	25	10
Participant 144	Ambulance Service D	36	Female	6	10
Participant 145	Ambulance Service D	59	Female	19	10
Participant 152	Ambulance Service G	44	Female	2	9
Participant 154	Ambulance Service G	27	Male	6	9
Participant 155	Ambulance Service B	51	Female	8	9
Participant 156	Ambulance Service B	21	Male	1	8
Participant 161	∼*		Non-binary	4	7.5
Participant 163	Ambulance Service B	46	Male	12	9
Participant 164	Ambulance Service B	27	Female	2	10

* Data available but omitted to protect confidentiality of this participant.

### Procedures

Ethical approval for the study was provided by the NHS, Invasive & Clinical Research Committee at the University of Stirling (Ref: 0539 25/02/2021). This, and approval by the Research and Development team at each site was obtained prior to approaching participants.

Eligible potential participants were identified by EMDC and invited to take part by email and via staff bulletins. Those interested in taking part contacted the researcher by telephone or email and were provided an opportunity to ask questions and give informed consent. Data collection only took place after written consent was obtained.

Semi-structured qualitative interviews were conducted with consenting call-handlers exploring their experiences of providing CPR instructions to callers, their perceptions of the most common barriers and the techniques they use to help people initiate CPR (see Supplementary File 1: Topic Guide). The topic guide was developed based on authors’ knowledge of the literature and designed to probe the under-explored area of call-handlers’ experiences.

Due to COVID-19 restrictions at the time, interviews were conducted via videoconferencing (Teams/Zoom as per participant preference). Interviews were audio-recorded with participants’ permission using an encrypted digital voice recorder, transcribed verbatim, anonymised and entered into NVivo v14 for coding. Participants received a £50 voucher payment to compensate them for their time.

### Analysis

Anonymised verbatim transcripts were coded by BF and RO using an adapted grounded theory approach to developing analysis A coding framework was developed and agreed drawing on a combination of *a priori* research questions, and inductively derived topics identified during familiarisation. Constant comparison, a continual process of critical reflection and comparison of data within and across interviews, was adopted.^35^, ^36^ This meant that each sentence or paragraph of every interview was initially examined and assessed as either being a good fit within the existing framework or as challenging our emerging theories about the data, requiring revision to the framework. Half of the interview transcripts were coded using the preliminary framework devised. Following discussion, and agreement between RO and BF, this was later refined and then applied to all transcripts. RO and BF also shared ‘analytic conversations’^37^ at the beginning of analysis, to compare their perceptions of these data, which helped guide attention to what both anticipated would be the most pertinent themes. BF focused on developing analysis of data relating to the barriers (and facilitators) to CPR. RO focused on data that provided a more deep and nuanced exploration into call-handlers’ experiences (some of which will be the subject of other reports).

## Findings

### Participants

Participants were recruited from seven ambulance services that ranged in size, geography and software used (see Table 1); reported rates of ROSC from 10% to 29% and CQC ratings from ‘requires improvement’ to ‘outstanding’. 30 call-handlers participated in qualitative interviews. Participants were aged 21–57; 20 were female, 9 male and 1 non-binary. They had between 6 months and 25 years of experience as a call-handler and participants self-reported confidence in providing CPR instructions ranged from 3/10 (in a participant with 6 months experience) to 10. At least two from each ambulance service and each country in the UK took part. Five participants used NHS Pathways and the remainder MPDS. (See Table 2).

### Frequency of CPR calls

Participants described CPR calls as being a regular but not frequent aspect of their work, reporting having provided CPR instructions from 0 to 9 times/day. Typically, call-handlers reported they expect to receive around one call per shift that required them to give CPR instructions but acknowledged it is unpredictable and varies widely.

*“Sometimes it feels like that’s all you get, like, you can just have a day with, like, CPR after CPR after CPR and then you can go for weeks without any.”* (P161, non-binary, 4 years’ experience).

### Barriers to CPR

Participants reported the proportion of CPR calls where they encountered barriers ranged from ‘not often’ to always. The wide variation may reflect differing experiences or alternatively differing thresholds for what constitutes a ‘hold-up’: “*depends on what you mean by hold up, I think probably every single one of them’s got a hold up of some point*” (P155, 51, female, 8 years’ experience) Most call-handlers reported hold-ups in most calls.

Four barriers to initiating CPR were identified by nearly all call-handlers: the strong emotions experienced by callers; physical issues; uncertainty about whether CPR was required, and caller concerns about doing harm. Other challenges identified by at least a third of participants included: where the person requiring CPR was a stranger to the caller; calls where lots of things were happening at once; COVID-related concerns; caller not processing information; caller being unable or uncooperative; traumatic arrests (i.e. cardiac arrests caused by external physical trauma rather than an underlying medical condition); system issues (e.g. not being able to locate shortcut to CPR instructions if arrest happens mid-call) and where rescuers were carers or from nursing homes.

### Emotion

Strong emotions were reported to hinder CPR initiation in a number of ways: call-handlers describe callers being unable to hear or listen to instructions (“*they're not listening to you, they can't hear you*” P145, 59, female, 19 years’ experience), or being slow to process information:

*“I was counting out compressions and I was like ‘there's no way she's doing these, I don’t think she's heard me, I don’t think any of this has gone in’ and I'm not even sure she's got the phone to her ear at this point because all I'm hearing is screaming and nothing”* (P105, 27, female, 1 year experience).

There were also examples where call-handlers reported being affected themselves by callers’ intense emotions, potentially affecting their ability to manage the call. Typically calls reporting suicides, paediatric deaths, and abusive callers, were perceived to be the most difficult, as the caller, particularly if a family member, are in a highly emotional state. Call-handlers described how callers would come on to the line “*hysterical*”, “*shouting*” (P105, 27, female, 1 year experience) and “*screaming (‘he’s dead, he’s dead)”* (P119, 31, female, 2 years’ experience). In these cases, call-handlers said they sometimes struggled to get the address of the incident, let alone progress to CPR (P110, 57, female, 8.5 years’ experience).

“*When I get a CPR call obviously the people calling me are normally very, very stressed, upset, manic, all sorts of colours of emotions as you can probably imagine*” (P163, 46, male, 1 years’ experience).

### Physical

Physical challenges included issues both relating to the rescuer, and to the patient. Regarding the rescuer, participants noted they were often older and with disabilities/medical conditions themselves. In relation to the patients, the most commonly mentioned issue was that heavy patients were particularly difficult to move “’*well I can't move him, I'm only nine stone and he's 15 stone*’” (P120, 53, female, 13.5 years’ experience). The situation people were in (e.g. wedged between furniture, in a car etc.) was also identified as causing difficulties. The vast majority of physical challenges were discussed in relation to the instruction to position the patient flat:

“*Navigating that patient off the bed is difficult a lot of the time. That’s the big one. And people in chairs, getting them onto the floor, especially with elderly callers…, yeah, getting people flat on their back on the floor is a real challenge*” (P105, 27, female, 1 years’ experience)

Some participants reported that physical limitations also impact the quality, and duration of, CPR: *“the person might say ‘look, I've a heart condition’ or ‘I have asthma’ you know, they may well have something wrong with them that might hinder them, maybe won't at least stop them doing CPR but it might hinder them to a degree where they'd maybe be able to do it but not be able to sustain it*” (P152, 44, female, 2 years’ experience).

## Uncertainty about whether CPR required

### Breathing or not?

Uncertainty about whether CPR was required was largely related to callers being unsure about whether or not the patient was breathing. Call-handlers identified that callers often mistook ineffective or agonal breathing for normal breathing, and this made it difficult to make the case for starting CPR. “*Sometimes they’ll be going ‘oh yeah they're breathing’ but in the background you can hear that it’s agonal”* (P101, 27, female, 4.5 years’ experience).

The assessment of breathing on the telephone was generally perceived to be fraught with problems. Several call-handlers mentioned that ‘breathing’ and ‘bleeding’ were often confused on the telephone, particularly when there was a language or communication barrier. Others said that most callers generally do not know how to tell if someone was breathing or not, making assessment of the patient’s condition difficult. Some were able to identify agonal breathing just by listening to the patient’s breath sounds in the background, though more inexperienced call-handlers identified challenges with this “*I think when you're new it can be easy to be persuaded by callers that the patient is breathing and so you spend a lot of time going round in circles*” (P105, 27, female, 1 year experience). The latter participant said they had been given feedback from audits that they had missed agonal breathing. A number of call-handlers also expressed that callers often struggled with responding to the breathing assessment tool used (this involves asking callers to say the word ‘now’ every time the patient takes a breath in so the call-handler can assess the breathing rate):

“*You say ‘tell me now every time he takes a breath, you say now’ and then they just stop. ‘Was he taking a breath?’, ‘yeah’, ‘but you need to keep saying now’ and oh, I mean, I've had times where I've started that tool, like, five times and I'm like ‘only stop when I tell you to stop’ and they're going ‘now, now’ and then they do something else and you're like ‘oh god’*” (P137, 36, female, 18.5 years’’ experience).

### Already dead?

Call-handlers also mentioned challenges and complexities around determining whether CPR was appropriate where the person might already be beyond help. Call-handlers described the precise, limited situations where they are instructed not to give CPR instructions (e.g. where patient is spontaneously described as cold and stiff in a warm room or in extreme circumstances such as decapitation) but reported that callers often say ‘*they’re gone*’ or ‘*he’s already dead*’ in circumstances where CPR could still possibly make a difference, causing a delay to getting CPR started:. “*Trying to tell someone to do CPR when they're flat out telling you ‘I'm not, I can't, he's dead, he's dead’ but we don’t know what's happened, and it’s not an obvious death… I find those are really hard*” (P133, 26, female, 1 year experience).

### Inappropriate to do CPR?

Additionally, call-handlers reported that callers mention concerns about whether to attempt CPR on elderly people “*you hear things like* ‘*oh I think their time is up now’*” (P123, 35, female, 8 years’ experience); people with terminal illness “*there's a bit of doubt as to whether CPR is the right thing to do for them by the caller, they might not know whether the patient wanted to be resuscitated, or even if they do have a Do Not Attempt (DNA) CPR’ in place sometimes*” (P103, 22, male, 1 year experience).

Ambiguous DNA CPR arrangements were frequently mentioned as causing confusion and delayed CPR, some participants highlighted this as a particular issue in relation to calls from care homes.

“*Cause most people in care homes have got DNARs but obviously we can't see that so we’re not in a position to say ‘no don’t do CPR’, it’s up to the crew when they get there if they’ve got it in their hand, but they* [care home staff] *normally don’t want to do it.”* (P164, 27, female, 2 years’ experience).

Calls from care/nursing homes and home-carers were described by some as relatively straightforward as carers ‘*not as emotionally involved*’ (P154, 27, male, 6 years’ experience) but others reported these calls to be regularly problematic describing a range of issues: some described carers (who may have known patient for a long period) as often being as distressed as family members (P155, 51, female, 8 years’ experience). Call-handlers expressed empathy (P113), recognising that many carers were young, inexperienced and it was a shock, but others noted some were surprisingly ‘*indifferent’* and unwilling to perform CPR. Some mentioned the widespread belief amongst carers that rules not to move patients who had fallen meant they couldn’t do CPR.

*“It’s hard to convince [them] that if their patient falls or is on the floor they're not allowed to pick them up. Now I'm telling you to drag them off the bed and crack their sternum while doing CPR*.” (P135, 32, male, 1 year experience)

Similarly, uncertainty from nursing staff about what to do surprised this call-handler who expected more from fully qualified health professionals “*I can't be needing to convince a healthcare professional the patient needs CPR. You need to be doing CPR before you've called me, you know better than me. I'm not medically trained, you are*” (P135, 32, male, 1 year experience).

### Facilitating factors

Interview questions were designed primarily to explore barriers to CPR (see Supplementary File 1), but some participants spontaneously mentioned facilitating factors. Four highlighted that child callers were generally easier to instruct to perform CPR, likely because they follow instructions less questioningly:

“*They're always incredible, amazing. Often I’ll say, like, you realise you're talking to a child and you'll say at some point ‘how old are you?’ and they're like ‘seven’ … and it’s incredible, it always makes me just, I’ll catch my breath …and that just shows to me that if you just followed the instructions and just answered the questions that you're being asked you wouldn’t have half of the problems that we do have*.” (P127, 52, female, 3 years’ experience)

The move to compression-only CPR amidst the COVID19 pandemic was perceived as having been helpful, both because it simplified the instructions call-handlers needed to give and because it eliminated reticence associated with performing rescue breaths.

Callers being ‘calm’ was mentioned as particularly helpful by a few, as was having multiple rescuers able to perform different roles (although managing multiple people at the scene can be problematic too).

## Discussion

Call-handlers from across the UK were remarkably consistent in identifying barriers to CPR, with almost all citing strong emotions, physical issues, uncertainty about whether CPR was required and caller concerns about doing harm as major issues. These are factors that have also been consistently identified in studies analysing call recordings^19^, ^21^, ^28^, ^23^, ^24^, ^25^, ^26^ and exploring the challenges of identifying cardiac arrest.^38^ Similarly, qualitative studies exploring the experiences of call-handlers, have previously highlighted emotion,^38^, ^39^, ^40^ physical issues,^39^ recognition of cardiac arrest^39^, ^41^ as challenging for call-handlers.

With evidence accumulating about the factors that most commonly impede DA-CPR, we suggest it is time to prepare call-handlers to overcome these specific barriers and to provide tools for them to do so. For example, training call-handlers in persuasive communication has been shown to reduce time to first compression and ROSC.^42^ Our team is exploring whether phrases with integrated behaviour change techniques specific to the most common barriers might help achieve faster CPR.

This study, added to the existing qualitative literature, does suggest that, despite the protocol-driven nature of the role, call-handlers quickly develop tacit knowledge. All CHs, without exception, said they considered CPR calls to be the most important calls they took. It is apparent, even within the limited sample of quotations in this paper, that call-handlers reflect on previous calls, develop an understanding of the caller perspective and recognise patterns in what gets in the way of CPR. Tapping into this valuable experiential knowledge is likely to be a fruitful method of identifying ways to improve DA-CPR and further efforts should be made to do so, in order to improve practice. Alongside this work to identify barriers, our team is also working on analysis of call-handlers’ tacit knowledge about the techniques they consider effective in achieving CPR.

The aggregate experience of our sample of call-handlers contained nuanced insights into the particular complexities associated with calls from care homes and from healthcare professionals, insights that may be more difficult to identify when samples of calls are selected for analysis. It may be beneficial for ambulance services to conduct additional focussed work to optimise processes for health and care professionals. Participants made clear in interviews that each call represents an intersection of many overlapping issues with the potential to delay CPR, making each a unique challenge, not always readily addressed by protocols. Call-handlers’ perspectives may be helpful in identifying priority issues for improving responses but many commented that they are rarely consulted about changes, a perception of being under-valued that has been observed by others.^40^, ^41^

## Strengths & limitations

A strength of this study is that it obtained rich, detailed accounts from a relatively large (in qualitative terms) and diverse group of call-handlers from across 7 ambulance services in the four countries of the UK. These data have enabled us to identify and explain call-handler’s perceptions of the barriers to CPR in a level of detail not previously reported. The use of participant quotes provides a rich, contextualised picture of how barriers are experienced by call-handlers.

However, there are also some limitations of this approach. Whilst call-handlers were encouraged to provide personal perspectives, drawn from their own experience, it is clear that some calls, such as those involving CPR, are frequently discussed between colleagues. This might influence the barriers call-handlers notice, meaning their responses might be subject to confirmation bias or reflect ‘group think’. The close alignment with results from studies with alternative methods (e.g. registry and retrospective analysis of call-recordings) provides support for their validity.

## Conclusion

Managing CPR calls is an important aspect of call-handlers role and responses indicate most are highly conscientious about trying to achieve CPR as quickly as possible. Call-handlers readily identified barriers to CPR (strong emotions, physical issues, uncertainty about whether CPR was required and caller concerns about doing harm) and were able to provide further detail about how these impact on time to CPR. Barriers identified are similar to those identified via call-recordings and registry data but in addition particular insights into complexities of ambiguous situations such as those encountered by carers and care-homes, DNACPR issues were identified. Call-handlers’ perspectives may be helpful in identifying priority areas for protocol refinement and ways to improve the efficacy of CPR instructions.

## CRediT authorship contribution statement

**Barbara Farquharson:** Writing – review & editing, Writing – original draft, Validation, Project administration, Methodology, Funding acquisition, Formal analysis, Data curation, Conceptualization. **Marie Johnston:** Writing – review & editing, Methodology, Funding acquisition, Conceptualization. **Rosaleen O’Brien:** Writing – review & editing, Formal analysis. **Gareth Clegg:** Writing – review & editing, Methodology, Funding acquisition, Conceptualization.

## Funding

This work was supported by the British Heart Foundation [grant number: FS/20/24/34944] and in part from the Laerdal Foundation

## Declaration of competing interest

The authors declare the following financial interests/personal relationships which may be considered as potential competing interests: Barbara Farquharson reports financial support was provided by British Heart Foundation. British Heart Foundation reports financial support was provided by Laerdal Foundation For Acute Medicine. If there are other authors, they declare that they have no known competing financial interests or personal relationships that could have appeared to influence the work reported in this paper.
