# Supplementary material for: “All sorts of colours of emotions”: Ambulance call-handlers’ perceptions of the barriers to CPR in out-of-hospital cardiac arrest
Source: Resusc Plus. 2025 Feb 15;22:100904. doi: 10.1016/j.resplu.2025.100904 (PMC11910351; doi:10.1016/j.resplu.2025.100904)
Supplement: Supplementary Data 1 [file mmc1.docx]

**Semi-structured Interview Schedule**

| **Welcome and introduction**   - Reiterate purpose of this qualitative interview (i.e. to explore participants’ views and experiences of providing t-CPR instructions) - Explain how the information they provide will be recorded, stored, transcribed and anonymised (e.g. pseudonym names, identifying information changed). - Advise participant that they can ‘pass’ on any question that they do not wish to answer. |
| --- |
| **The following areas will be covered within the interview process:** |
| **1. Your experiences of your role as someone who provides t-CPR** |
| - How often in an average shift are you required to give t-CPR instructions? - How do you feel about this element of your job? - What do you find difficult about these calls? - What do you enjoy about these calls? - How do calls involving t-CPR compare with the rest of your work? - How were you prepared for this aspect of your work? What training did you receive? How well did that preparation help you? Is there anything you feel would have prepared you better? - What support do you receive at work in relation to t-CPR? How helpful do you find that? Is there anything that isn’t currently provided that might help? |
| **2. Barriers to t-CPR** |
| - How often do you encounter hold ups in getting someone to perform CPR? - What are the main things that prevent rescuers providing CPR? - Which things do you find the hardest to deal with? - What are your ‘tricks of the trade’ for overcoming these barriers? What sort of things do you try [for each barrier identified] and what do you think works best? - Would you like to tell me about a t-CPR call that you felt you made a real difference in? - And one where you wish you’d done things differently? - Are any of your colleagues particularly good at t-CPR? What makes them so good do you think? - What have you learned along the way that has helped with this aspect of your job? How did you learn that? - We’re thinking about adapting some elements of the MPDS protocol to include some psychological techniques that might help people initiate CPR more quickly – what are your thoughts about that? |
| **3. Anything else we need to know?** |
| - If we were keen to increase the number of people doing CPR in OHCA is there anything else we need to know from your point of view? |
| **Thank you/Offer opportunity to ask questions/de-brief** |
